# Feasibility of non-invasive Foetal electrocardiography in a twin pregnancy

**DOI:** 10.1186/s12884-020-02918-8

**Published:** 2020-04-15

**Authors:** Lore Noben, Michelle E. M. H. Westerhuis, Judith O. E. H. van Laar, René D. Kok, S. Guid Oei, Chris H. L. Peters, Rik Vullings

**Affiliations:** 1 Department of Obstetrics and Gynaecology, Máxima Medical Centre, P.O. Box 7777, 5500 MB Veldhoven, The Netherlands; 2 Eindhoven MedTech Innovation Centre (e/MTIC), P.O. Box 513, 5600 MB Eindhoven, The Netherlands; 3 Nemo Healthcare BV, ‘MMC Incubator’, De Run 4630, 5504 DB Veldhoven, The Netherlands; 4 Department of Clinical Physics, Jeroen Bosch Hospital, P.O. Box 90153, 5200 ME, ‘s Hertogenbosch, The Netherlands; 5 Department of Electrical Engineering, Eindhoven University of Technology, P.O. Box 513, 5600 MB Eindhoven, The Netherlands

**Keywords:** Foetal monitoring, Foetal electrocardiogram, Foetal heart rate, Twin pregnancy, Multiple gestation

## Abstract

**Background:**

Twin pregnancy is associated with increased perinatal mortality. Close foetal monitoring is therefore warranted. Doppler Ultrasound cardiotocography is currently the only available method to monitor both individual foetuses. Unfortunately, the performance measures of this method are poor and erroneous monitoring of the same twin with both transducers may occur, leaving the second twin unmonitored. In this study we aimed to determine the feasibility of monitoring both foetuses simultaneously in twin gestation by means of non-invasive foetal electrocardiography (NI-fECG), using an electrode patch on the maternal abdomen.

**Methods:**

A NI-fECG recording was performed at 25 + 3 weeks of gestation on a multiparous woman pregnant with dichorionic diamniotic twins. An electrode patch consisting of eight adhesive electrodes was applied on the maternal abdomen, yielding six channels of bipolar electrophysiological measurements. The output was digitized and stored for offline processing. The recorded signals were preprocessed by suppression of high-frequency noise, baseline wander, and powerline interference. Secondly, the maternal ECG was subtracted and segmentation into individual ECG complexes was performed. Finally, ensemble averaging of these individual ECG complexes was performed to suppress interferences.

**Results:**

Six different recordings were obtained from each of the six recording channels. Depending on the orientation and distance of the fetal heart with respect to each electrode, a distinction could be made between each fetus based on the morphology of the signals. Yielding of the fetal ECGs was performed manually based on the QRS complexes of each fetus.

**Conclusion:**

NI-fECG with multiple electrodes allows for monitoring of the fetal heart rate and ECG of both individual fetuses in twin pregnancies.

## Background

Multiple gestation is the most common high-risk condition in obstetric medicine, with a varying incidence from 6.7 per 1000 births in Japan to 40 per 1000 births in Nigeria [1]. In the Netherlands, 1.6% of all deliveries after 22 weeks of gestation in 2017 were twins [2]. Twin pregnancies are associated with increased perinatal morbidity and mortality rates [3]. They have a higher risk of foetal growth restriction and preterm birth compared to singleton pregnancies [4]. In monochorionic twin pregnancies, both twin-twin transfusion syndrome (TTTS) and twin anaemia polycythaemia sequence (TAPS) are complications which can result in foetal death when left untreated [5]. Hence, close foetal surveillance is imperative for early identification of complications and intervention. As in singleton pregnancies, foetal monitoring in twin pregnancies is done by means of the cardiotocogram (CTG). The non-invasive version of this method consists of a tocodynamometer (TOCO) which registers uterine activity and a Doppler ultrasound (DU) transducer to obtain the foetal heart rate (FHR). In case of multiple pregnancies, each foetus requires its own DU transducer. Erroneous monitoring of the same twin with both transducers may occur. The observation of identical tracings can avoid fatal consequences in this situation, such as foetal demise due to undiagnosed chronic hypoxia. Unfortunately, DU CTG has a poor specificity and high inter- and intra-observer variability, since it is dependent on visual assessment by a physician [6]. Furthermore, DU CTG is highly sensitive to signal loss due to foetal and maternal movement and its performance is negatively correlated with the maternal BMI [7]. The fixating elastic bands may cause discomfort for the pregnant woman while the need for multiple DU transducers in multiple gestation often requires more elastic bands. The CTG can also be obtained via invasive means: the foetal scalp electrode (FSE) is the gold standard for foetal monitoring. However, this invasive method can only be applied once membranes have ruptured and with sufficient cervical dilatation. Therefore, this method is only suitable during labour and not for antepartum monitoring. Furthermore, invasive CTG has a higher risk of injury and infection and only registers the FHR of the leading twin. Monitoring of the FHR with a DU transducer is still required for the second twin.

For evaluation of the FHR pattern, simultaneous registration of uterine activity is required. The intra-uterine pressure catheter (IUPC) is an invasive technique which is considered the gold standard for contraction monitoring. Due to the reported risks of placental and uterine perforation, TOCO is the current method of choice [8, 9]. However its performance is negatively influenced by maternal BMI and often needs to be relocated due to maternal movements [10].

Non-invasive foetal electrocardiography (NI-fECG) uses multiple electrodes, possibly combined in a single patch, on the maternal abdomen to monitor both foetal and maternal heart rate as well as uterine contractions by means of the electrohysterogram (EHG). This method was first described as early as 1906 [11]. Due to the technical struggle of isolating the low voltage foetal signal from the relative large maternal signal, development of the technology lagged behind the development of DU and FSE technology. Recent advances in signal processing techniques made it possible to separate the FHR from the interfering signals. Taylor et al. previously described the use of NI-fECG technology to separate both individual foetal signals in twin pregnancies, using twelve lead electrodes [12].

In this paper we present a case of a twin pregnancy in which successful separation and differentiation of both foetal signals was achieved, measured with one single electrode patch consisting of only 6 electrodes on the maternal abdomen. Based on the ECG of each individual foetus, a continuous FHR trace can be monitored and plotted.

## Methods

**Fig. 1:**
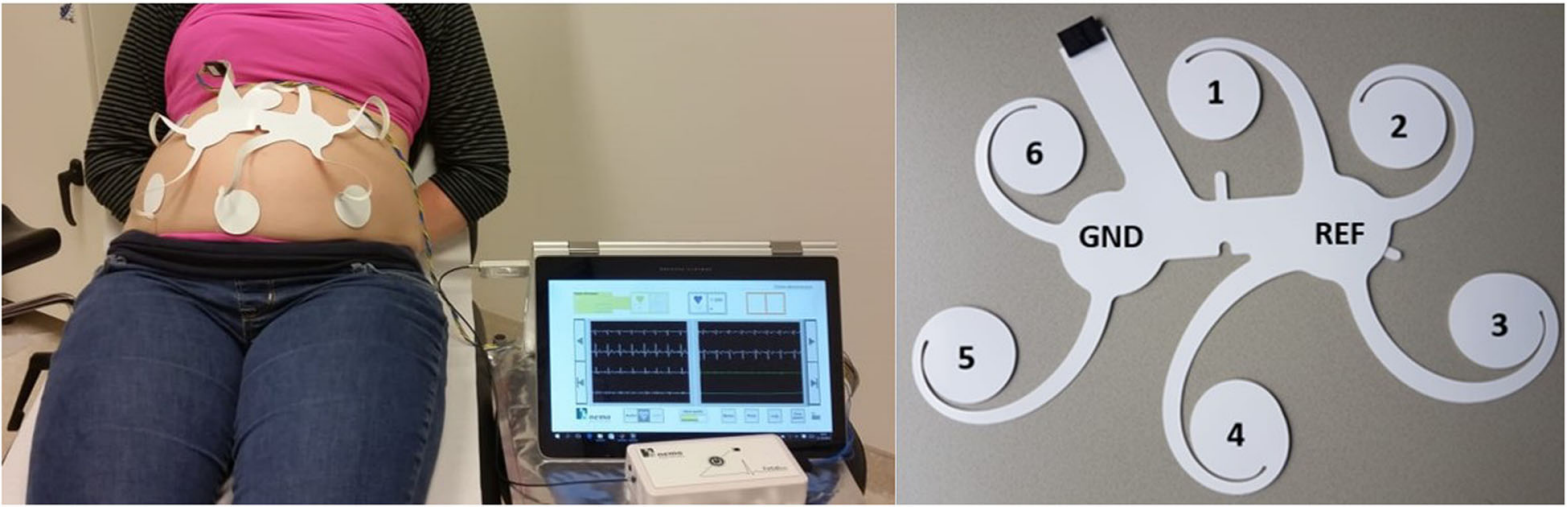
Illustration of the non-invasive foetal electrocardiography (NI-fECG) device and electrode patch. The left picture shows the electrode patch applied on the maternal abdomen and attached to the amplifier and a computer. The right picture shows the electrode patch with the numbered electrode channels and the ground (GND) and reference (REF) electrode

A 33-year old gravida 2, para 1 with dichorionic diamniotic twins received a one-time foetal ECG recording at 25 + 3 weeks of gestation in a research context after written informed consent was obtained. We received a statement of the Institutional Review Board of the Máxima Medical Centre stating that no ethical approval was required (N18.074). She previously delivered a healthy female neonate at term gestation, weighing 3460 g. Fetal anomaly screening at 20 weeks of gestation showed no abnormalities in both foetuses. The foetal ECG was recorded with six channels, using a prototype of the Nemo non-invasive electrophysiological monitoring device (Nemo Healthcare BV, Veldhoven, the Netherlands). An electrode patch was placed on the maternal abdomen consisting of 8 electrodes, including one ground and one reference electrode (Fig. 1), yielding six channels of bipolar electrophysiological measurements. Before applying the patch, the skin was washed with water and soap and prepared using medical abrasive paper (Red DotTM Trace Prep, 3 M Health Care, Canada). The recording lasted for 28 min, during which the pregnant woman was lying in a semi-recumbent position to prevent aorta-caval compression.

The recording was digitized at 500 Hz and stored for offline processing. Offline data analysis was performed using MATLAB® (The MathWorks, Inc., MA, USA). The recorded signals were first pre-processed by suppression of high-frequency noise, baseline wander, and powerline interference. Then, the maternal ECG was suppressed using a dynamic template subtraction technique [13]. Two linear combinations of the six recorded signals were generated to separate both foetuses ECG signals. Each combination enhanced the foetal ECG signal for one twin, while suppressing the ECG for another. The enhanced foetal ECG signals were used to detect the location of foetal QRS complexes. These locations served as reference to perform segmentation of individual ECG complexes for each of the six recorded signals of each foetus. Subsequently, ensemble averaging (i.e. averaging the ECG complexes over all heartbeats) of these individual ECG complexes was performed to further suppress interferences. It should be noted here that ensemble averaging for one twin means suppressing the ECG of the other foetus. The result of these signal processing steps is an average foetal ECG complex for each of the six recorded channels for each of the foetus. Finally, knowledge on the locations of the recording electrodes was used to calculate a foetal vectorcardiogram for each twin [14].

The shape of the calculated foetal vectorcardiogram depends on the orientation of the foetus within the uterus. A foetus in cephalic orientation should have a vectorcardiogram that is rotated by 180 degrees compared to a foetus in breech presentation. Assuming both twins to have a normal vectorcardiogram with normal electrical heart axis the orientation of the foetus within the uterus can be estimated [14, 15]. This estimation was blinded from the orientations that were determined by ultrasonic examination during the measurement.

## Results

**Fig. 2:**
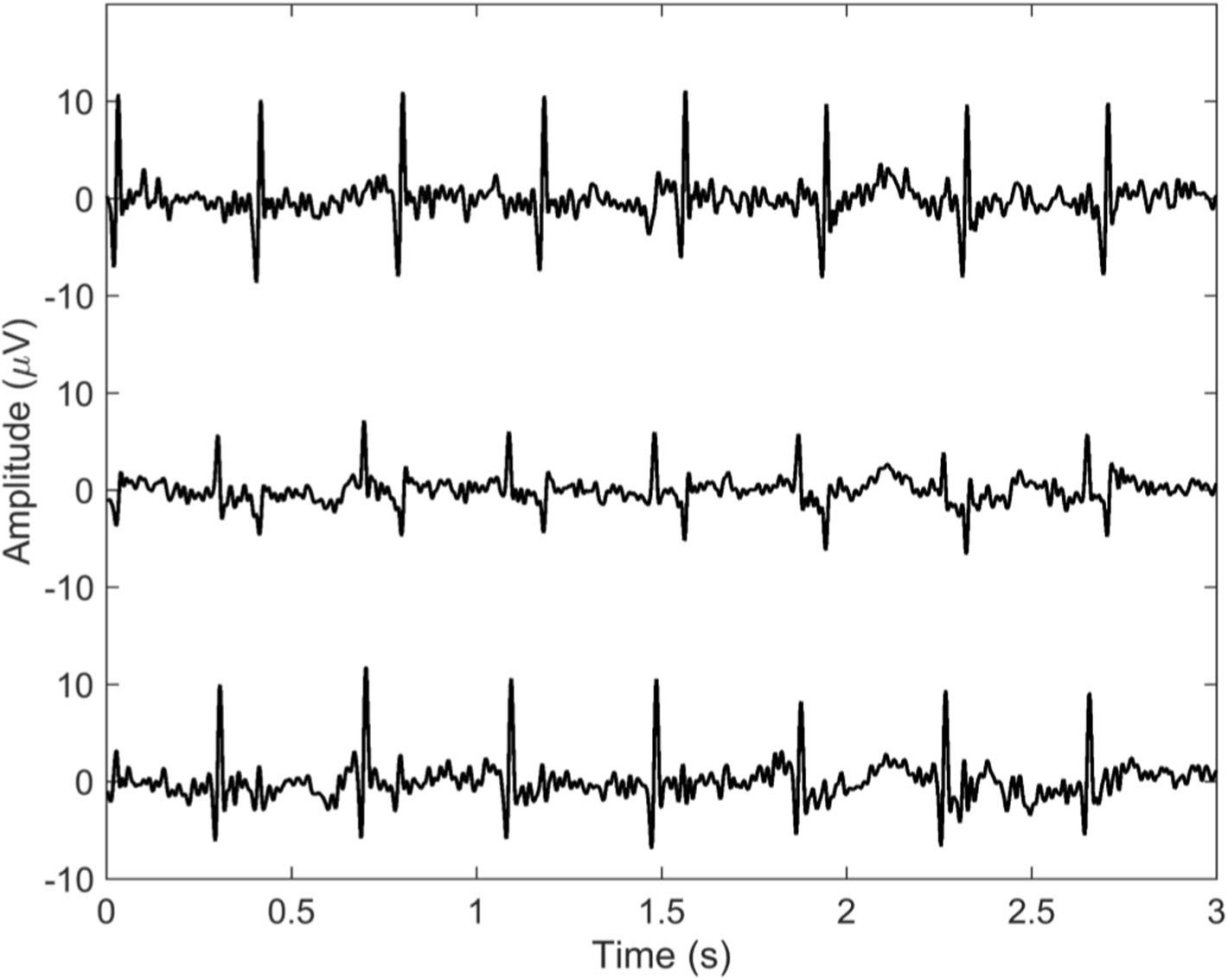
Foetal signals obtained from electrode number 3 (top graph), electrode number 4 (middle graph) and electrode number 5 (bottom graph) as numbered in Fig. 1. The top graph comprises the electrocardiogram (ECG) of the foetus located in utero on the right, beneath electrode number 3. The bottom graph comprises the ECG of the foetus located in utero in the left, beneath electrode number 5. The middle graph contains the ECGs of both foetuses, derived from electrode number 4, which was situated in the midline

Figure 2 shows the foetal signals obtained after suppression of the maternal ECG from the bottom three electrodes. Based on these ECGs it is possible to differentiate both foetuses with respect to each electrode. The top graph (Fig. 2) comprises the ECG derived from electrode number 3 (Fig. 1). This ECG shows mainly QRS complexes of the foetus positioned on the right side of the uterus, in proximity to this electrode. The bottom graph (Fig. 2) comprises the ECG of the foetus located on the left, derived from electrode number 5 which is positioned on the left side of the maternal abdomen (Fig. 1). Electrode number 4 picks up signals from both foetuses, since this electrode was located around the midline in between both foetuses (Fig. 1). The middle graph therefore contains QRS complexes of both foetuses, one with clearly positive QRS deflections, one with negative deflections.

**Fig. 3:**
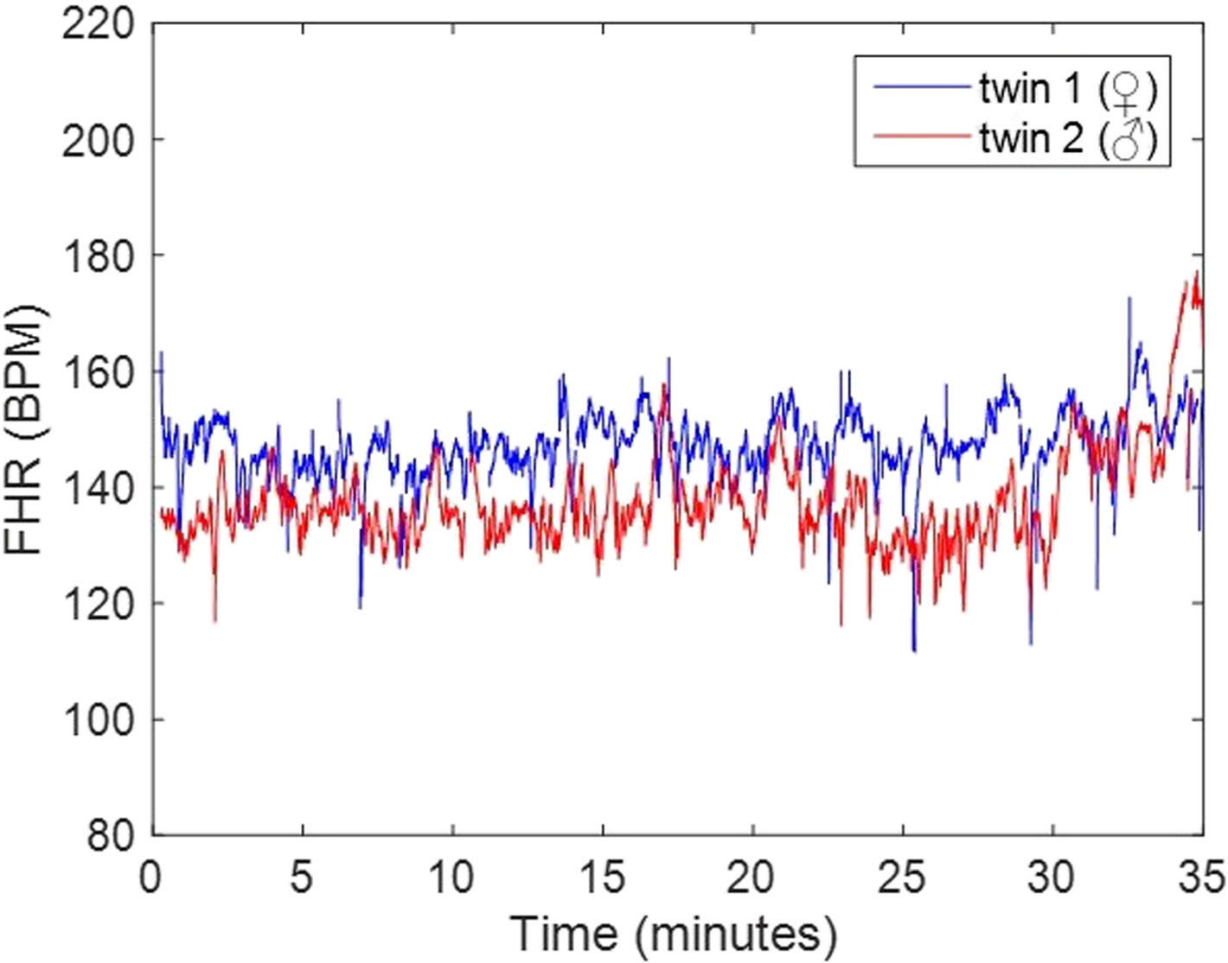
Continuous tracing of beat-to-beat foetal heart rate of both individual foetuses, based on the QRS-complexes of the foetal ECG, which resembles the display of a FHR tracing monitored with the widely used Doppler ultrasound cardiotocography

**Fig. 4:**
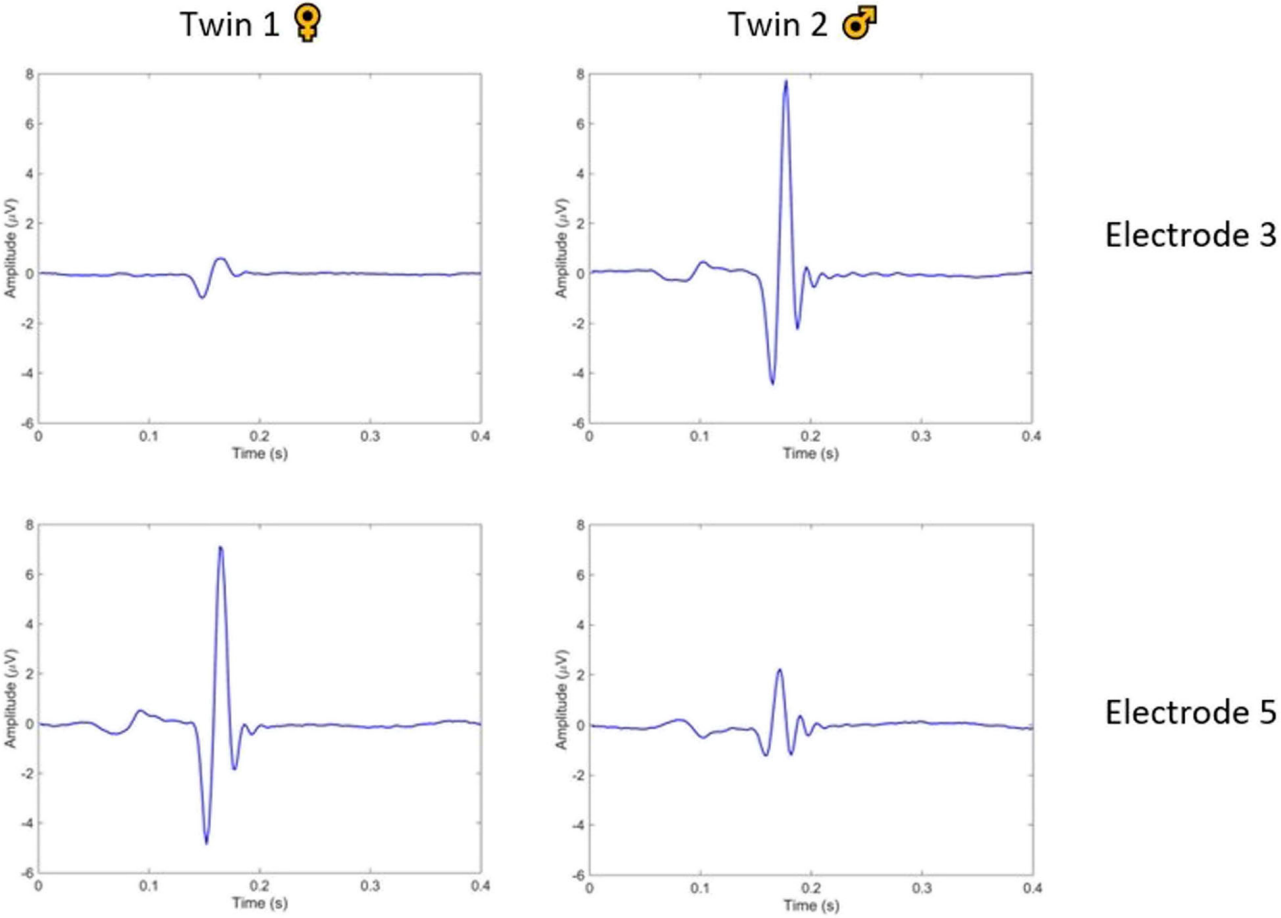
Average foetal ECG complex for both foetuses derived from the two outer electrodes, by means of ensemble averaging based on the QRS locations of each foetus

After differentiating the foetal ECG signals from both foetuses, beat-to-beat foetal heart rate can be calculated based on the detected foetal QRS-complexes and plotted as a continuous FHR trace for clinical practice (Fig. 3). Moreover, an average foetal ECG complex per foetus was yielded by means of ensemble averaging based on the QRS locations of each foetus (Fig. 4).

**Fig. 5:**
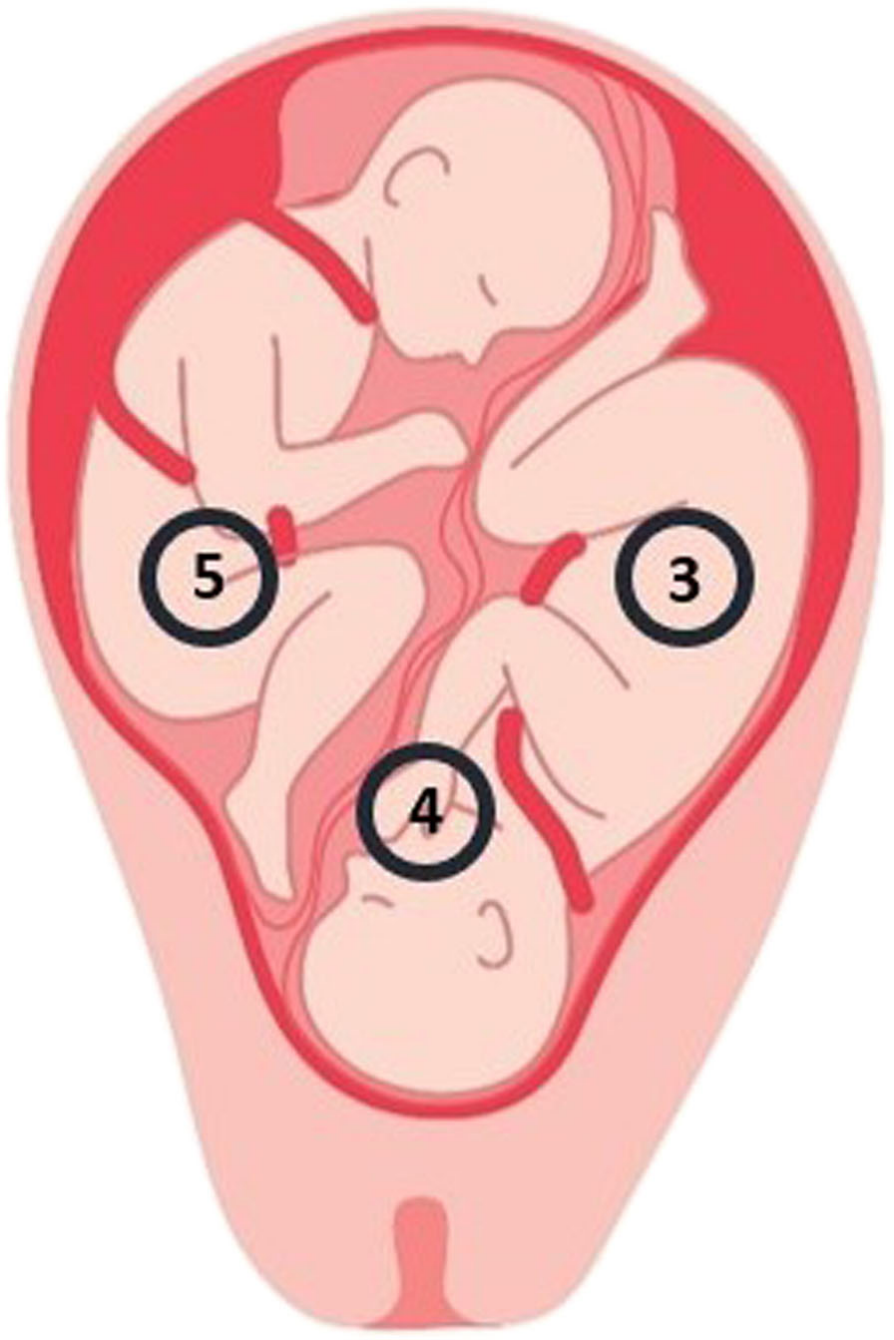
Representation of the position of both foetuses in utero during the measurement, estimated based on both vectorcardiograms. The female foetus is positioned on the left side of the image in breech presentation (Twin 1). The male foetus is positioned on the right side of the image in cephalic presentation (Twin 2).

Finally, a foetal vectorcardiogram was calculated for each foetus of which the orientation of both foetuses was estimated (Fig. 5). These orientations were confirmed by ultrasonic examination during the measurement.

## Discussion

We have demonstrated that non-invasive foetal electrocardiography is feasible for simultaneous FHR monitoring of both foetuses in twin pregnancies. Taylor et al. [12] first described successful separation of individual foetuses in multiple gestation using the NI-fECG. They used 16 electrodes to obtain five-minute recordings from both twin and triplet gestations. In 42 of 58 (72%) twin gestations, separation of both foetuses was possible [12].

In our case, separation of the foetal ECG signals from both twins was done manually. According to the ECG principle as in adults, electrical activity towards the electrode causes a positive deflection [16]. While in adults the electrodes are placed in a fixed configuration relative to the cardiac mass, the foetus can move around freely in the uterus. In twin pregnancies, the positions, orientations and distance from each foetal heart relative to each electrode is differed. This led to the different waveform amplitudes and morphology of both foetal ECGs derived from the different electrodes. Based on how the patterns of both foetal ECGs vary across the different electrodes, a vectorcardiogram can be calculated for each foetus. The vectorcardiogram is a three-dimensional representation of the electrical activity during one cardiac cycle from which the foetal orientation can be estimated, under the assumption of a healthy, normal heart [17].

The use of NI-fECG for foetal monitoring offers many diagnostic opportunities. Since it delivers beat-to-beat information on the FHR, based on the QRS-complexes of the foetal ECG, foetal heart rate variability of both foetuses can be analysed through spectral analysis [18–20]. This could aid in the diagnosis and surveillance of TTTS in monochorionic twin pregnancies, but also pre-eclampsia and foetal growth restriction, which are more common in multiple gestation [21]. Furthermore, the possibility of obtaining an (averaged) foetal ECG complex facilitates the detection of changes in ECG waveform, which can provide valuable information about the foetal condition in the antenatal period as well as during labour. Velayo et al. [22] previously described the use of foetal ECG parameters to differentiate the donor and recipient foetus in monochorionic pregnancies complicated with severe TTTS from non-TTTS monochorionic or singleton pregnancies. Their findings reflected cardiac dysfunction in the recipient twin due to the increased cardiac output [22].

Moreover, changes in the ST-segment of the foetal ECG are related to metabolic acidosis of the foetus [23]. Consequently ST-segment analysis (STAN, Neoventa Medical AB, Mölndal, Sweden) was introduced at the end of the twentieth century as a promising tool to detect impending metabolic acidosis during labour [23]. Unfortunately, the initial beneficial effect of STAN on perinatal outcome could not be confirmed in subsequent studies [24, 25]. Previous research has shown that variation in the orientation of the electrical heart axis between foetuses causes different T/QRS baseline values. Foetuses with a higher T/QRS baseline value were shown to be more prone to false positive ST events [26, 27]. Multi-lead NI-fECG recordings can deliver a 12-lead foetal ECG, taking information on the orientation of the electrical heart axis, derived from ultrasound evaluation, into account. This is in contrast to STAN, in which the signals are obtained during labour from a single-lead (FSE) and therefore can only be applied to the leading twin.

Although separation of the foetal ECGs of the twins was performed manually in this case, we expect that computerized separation of both twins is feasible, for instance using (blind) source separation techniques [28]. This would allow for real-time monitoring of the FHR in twin gestations, delivering a continuous heart rate tracing similar to that of the currently used Doppler ultrasound (Fig. 3) but without the risk of confusion of both foetal heart rates. Since the electrode patch also registers the maternal heart rate as well as uterine activity, it is a beneficial method for foetal monitoring in twin gestation, where there is a 12-fold higher rate of preterm birth [29]. Further research towards incorporating computerized separation techniques and using multiple twin pregnancies to test the reproducibility is needed before this technology can be implemented in clinical practice.

## Conclusion

Our research shows that in a twin pregnancy non-invasive electrophysiological foetal ECG recording with multiple electrodes allows for monitoring of the FHR and ECG of both individual foetuses. This technology may introduce an alternative method for non-invasive foetal monitoring in twin pregnancies, after further enhancement of the signal separation techniques.

## Data Availability

The datasets used and/or analysed during the current study are available from the corresponding author on reasonable request.

## Abbreviations

NI-fECG: Non-invasive foetal electrocardiography
ECG: Electrocardiogram.
CTG: Cardiotocography.
DU: Doppler ultrasound.
FHR: Foetal Heart rate.
